# New Appliances and Things Medical

**Published:** 1903-03-21

**Authors:** 


### NEW APPLIANCES AND THINGS MEDICAL.

[We shall be glad to receive at our Office, 28 & 29 Southampton Street, Strand, London, W.C., from the manufacturers, specimens of all new preparations and appliances which may be brought out from time to time.]

### MARMITE.

(The Marmite Food Extract Company Limited, 40 Mincing Lane, London, E.C.)

This preparation is a new food extract prepared from vegetables: it makes delicious and stimulating bouillon and is a most valuable addition to soups and gravies, strengthening them and imparting flavour. As compared to the ordinary meat extracts which are usually employed for the above purposes it has certain distinct advantages: for instance, extractives derived from meat are not always indicated in gouty and rheumatic conditions, whereas vegetable extractives may be more safely employed and their stimulating properties are of nearly the same value. We therefore regard Marmite as likely to prove of great value in the treatment of the sick. Chemical analysis shows that Marmite contains about 25 per cent. of nitrogenous bases, and more than 10 per cent. of peptones and albumoses. Since the latter must be regarded as foods, this new preparation has nutritive as well as stimulating properties. In addition to these advantages it is very agreeable to the palate and very economical in use, since it is a cheap and highly concentrated extract.

### DRIESSEN'S COCOA POWDER.

(A. Driessen, Rotterdam. British Agent: De Beer Brothers and Co., 28 Minories, London, E.C.)

We have examined this cocoa powder and regard it as a very pure preparation, highly soluble, and free from the objectionable chemical bodies which are sometimes employed for securing a high degree of solubility. It is highly concentrated, a small teaspoonful of the powder being sufficient to make a large cup of cocoa. Its purity, delicacy of flavour, and the facility with which the beverage may be prepared render Driessen's Cocoa a reliable and valuable preparation.

### THROGMORTON SCOTCH WHISKY.

(J. Lyons and Co., Ltd., Cadby Hall, Kensington, London, W.)

This very excellent brand of whisky is guaranteed to consist of pure all-malt spirit with an average maturity of eight years. As far as chemical analysis can prove the accuracy of the above statement, our examination entirely bears out these claims. We find this whisky to be an exceedingly mellow spirit, of fine flavour, and such as we should confidently recommend for those invalids who require alcoholic stimulants.

